# Editorial: The role of GABA-shift in neurodevelopment and psychiatric disorders

**DOI:** 10.3389/fnmol.2023.1162689

**Published:** 2023-02-28

**Authors:** Lorenz S. Neuwirth, Abdeslem El Idrissi, Atsuo Fukuda, Werner Kilb

**Affiliations:** ^1^Department of Psychology, SUNY Old Westbury, Old Westbury, NY, United States; ^2^SUNY Neuroscience Research Institute, SUNY Old Westbury, Old Westbury, NY, United States; ^3^Center for Developmental Neuroscience, The College of Staten Island (CUNY), Staten Island, NY, United States; ^4^Department of Biology, The College of Staten Island (CUNY), Staten Island, NY, United States; ^5^Department of Neurophysiology, Hamamatsu University School of Medicine, Hamamatsu, Japan; ^6^Institute of Physiology, University Medical Center, Johannes Gutenberg University, Mainz, Germany

**Keywords:** GABA, GABA_A_ receptor (GABA_A_R), KCC2, NKCC1, Cl^−^ homeostasis

The main inhibitory neurotransmitter in the mature brain, GABA, has the unique feature that post-synaptic responses upon it's interaction with GABA_A_ receptors can change their direction in response to alterations in the intracellular Cl^−^ concentration. This GABA-shift plays an important role during early neurodevelopment and has been implicated in a variety of neuropathological conditions. The mechanisms behind the GABA-shift are mediated by changes in the expression and functions of key transporters for Cl^−^ and bicarbonate, in particular, Cl^−^ transporters NKCC1 (i.e., mediating Cl^−^ import) and KCC2 (i.e., mediating Cl^−^ extrusion). The current Research Topic sought to provide a forum for reviewing recent progress in this fascinating field and to collect recent studies investigating the role of the GABA-shift in neurodevelopment and in the etiology of neurological diseases.

The current Research Topic comprises five review articles and seven original research articles. The review articles provide an update on features of the GABA-shift, spanning from the structural basis of its regulation, *via* its role during neurodevelopment up to new perspectives for the etiology and treatment of neurological disorders. The original research articles focus on the role of Cl^−^ homeostasis under physiological conditions or in neurological disorders and present new experimental methods to determine the reversal potential of GABA.

A thorough introduction into the molecular basis of Cl^−^ homeostasis and regulation is provided by the review article of Hartmann and Nothwang, which provides an update on the structural basis underlying the regulation of KCC2 and NKCC1. The authors characterize phosphorylation sites on both transporters and describe the functional consequences of phosphorylation/dephosphorylation at these sites. They conclude that the intracellular loop between the α8 and α9 helix represents a region of particular importance for the functional regulation of KCC2. The review article by Kilb emphasizes that depolarizing GABAergic responses are not excitatory *per se* and provides a theoretical framework and experimental findings determining whether GABAergic depolarizations are inhibitory or excitatory. A more global review on GABAergic neurodevelopment is provided by Warm et al., summarizing the role of GABAergic interneuron populations in the functional maturation of the cerebral cortex and further describing the mutual interaction between maturation of the GABAergic system and cortical network activity. The remaining two review articles focus on the role of the GABA-shift in neurological and neuropsychiatric diseases. Hui et al. discusses how the developmental shift from excitatory-to-inhibitory GABAergic actions is altered in neurodevelopmental and neuropsychiatric disorders. They concentrate on the cell signaling and regulatory mechanisms underlying this GABA-shift and discuss how the GABA-shift influences interactions between GABAergic interneurons and other cell types in the developing brain and thereby contributes to neurodevelopment. Finally, they briefly outline recent progress on targeting NKCC1 and KCC2 as a therapeutic strategy against neurodevelopmental and neuropsychiatric disorders. More specifically, Huang et al. concentrated on the role of the GABAergic system in post-traumatic stress disorders (PSTD) and reviewed changes of the GABAergic system in PTSD based on imaging and pharmacological results from both preclinical and clinical studies and derived putative pharmacological targets that might be helpful in the future treatment of PTSD.

The original research article of Vazetdinova et al. reported the accuracy of cell-attached recordings to determine important cellular physiological parameters, which established that cell-attached recordings of cortical and hippocampal neurons can be used to reliably determine the GABA reversal potential and other physiologically relevant parameters. Therefore, cell-attached recordings can be used to investigate the GABA-shift, because they don't artificially disturb the intracellular Cl^−^ concentration. Two original research reports deal with the role of hyperpolarized GABAergic responses in GABAergic interneurons for the control of cortical excitability. Zavalin et al. reported that depletion of KCC2 in Dlx5-lineage neurons, which targets several types of GABAergic interneurons including parvalbumin-positive interneurons (PV-INs), induces a massive change in the distribution of GABA interneuron subpopulations, a high incidence of spontaneous seizures, and a high rate of premature death in juvenile animals. In contrast to their initial hypothesis, they did not observe migration deficits or disturbed laminar organization of interneurons, indicating that the adverse effects should occur later. Alternatively, they observed a milder phenotype in mice if KCC2 expression was obsolete only in PV-INs. In line with this, Herrmann et al. reported that a Cre-mediated disruption of KCC2 specifically in PV-INs led to the expected shift in the GABA reversal potential and a higher frequency of inhibitory post-synaptic potentials, indicating a disinhibition of PV-INs. In addition, these animals displayed a reduced seizure threshold with the occurrence of increased spontaneous seizures and an upregulation of pro-apoptotic genes in parvalbumin-positive interneurons.

At several locations in the adult brain, the developmental GABA-shift does not occur and GABA maintains a depolarizing action until adulthood. One of these regions is the hypothalamic medial eminence, involved in the hypothalamic-pituitary-adrenal (HPA) axis of corticosterone release. Yesmin et al. investigated the GABAergic network in this area and observed that a subpopulation of GABAergic neurons directly project to the axon terminals from CRH neurons. The conditional deletion of NKCC1 from the CRH axon terminals results in significantly lower corticosterone levels, demonstrating the important role of depolarizing GABAergic responses in the HPA axis and may serve as an early pathological trigger for later psychiatric issues.

Three additional articles revealed that modification of GABAergic system elements can led to persisting alterations in the excitability unrestricted to the GABAergic system. Sinha et al. reported that a depletion of WNK3, a developmentally expressed member of the WNK-family that regulates Cl^−^ homeostasis *via* phosphorylation of NKCC1 and KCC2, results in slightly higher intracellular Cl- concentrations. However, the authors observed that other neuronal properties determining excitability (e.g., K^+^-channel expression) are also altered in the WNK3 knockout animals, but that this effect can be ameliorated by an overexpression of KCC2, suggesting that the interactive function of WNK3 is probably the maintenance and development of both intrinsic and synaptic excitabilities. A comparable interaction between the GABAergic synaptic system and intrinsic neuronal excitability was reported by Hosoi et al., who investigated the role of taurine, an important agonist of the GABAergic system during early development and a modulator of WNK and hence Cl^−^ homeostasis, on the excitability of matured neurons. They report an obvious alteration of firing properties in differentiated Layer 2/3 pyramidal neurons of taurine-depleted animals. Lastly, Qin et al. discloses that inhibition of σ1- receptors, an orphan-receptor associated to depression-like phenotypes, reduces the expression of the GABA_A_ receptor subunits α1, α2, β2, and β3 in the nucleus accumbens. The resulting reduction in GABAergic transmission contributes to the impaired long-term depression and a depression-like phenotype in these mice.

In summary, the current Research Topic summarizes recent opinions on advancing our understanding of the mechanisms and functional consequences of the GABA-shift from the last three decades, but also provides evidence that changes in the GABAergic responses, either acute or during early developmental stages, can lead to persistent functional changes in the nervous system by affecting the GABA-shift and other subsequent processes that move beyond the GABAergic system completing the full integration of the mature brain.

## Author contributions

All authors listed have made a substantial, direct, and intellectual contribution to the work and approved it for publication.

